# Correspondence

**Published:** 1902-04

**Authors:** 


### CORRESPONDENCE.

### DISCUSSION OF "COLUMELLA'S" PROPOSITION.

EDITOR OF THE JOURNAL OF COMPARATIVE MEDICINE, ETC.:

"COLUMELLA'S" plan for disposing of tuberculous cattle, outlined in THE JOURNAL OF COMPARATIVE MEDICINE, ETC., vol. xxiii., No. 1, appears to me to be a very good one. I believe that Pennsylvania, being a State in which more has been done than in any other to eradicate tuberculosis, is now ready for the adoption of this plan. I notice by the annual report of the State Veterinarian, Dr. Leonard Pearson, that there are on file in his office more applications for tuberculin tests of herds than can be granted on account of lack of funds. If this economical plan of disposing of the tuberculous cattle could be used instead of paying for diseased cattle out of the State appropriation, this money could be devoted entirely to testing cattle. In this way all the applications could be granted; also, the ability to secure better values than the State pays for tuberculous cattle would lead more and more herd owners to apply for tuberculin tests for their herds, and there would not be the necessity of refusing to grant these tests on account of deficiency in funds, even should the appropriation stand at the present sum of $40,000. In this way tuberculin testing of herds would finally become a universal practice in that State, and there would be speedy identification of all the tuberculous cattle in the State, which could be put in the care of licensees. I do not know of another State which is so well prepared for the plan of "Columella" as Pennsylvania. If Pennsylvania should demonstrate the feasibility of this plan the other States would probably soon follow its example.

There would probably be some irregularities in the handling of the tuberculous cattle on account of dishonesty of licensees, but these could soon be detected and rectified. Certainly the fear of an occasional violation should not deter us from adopting what seem to be salutary laws.

The improvement which the method makes over the Bang method lies in the fact that an owner can handle properly a herd of cattle all of which are tuberculous much easier than an owner can handle properly two herds, one of which is healthy and the other tuberculous. This step of creating licensees is the only one necessary to make the Pennsylvania plan the ideal one for the eradication of tuberculosis.

JOHN J. REPP,
Veterinarian to the Experiment Station, Ames, Iowa.

---

EDITOR OF THE JOURNAL OF COMPARATIVE MEDICINE, ETC.:

Regarding the "Columella" proposition for combating tuberculosis, my absence from home is the reason for so tardy a reply.

The proposition seems to me a very good one and worthy of notice. I have had considerable experience with this disease, and am frank enough to say we are not getting enough benefit for the money expended. No doubt there are some little details in "Columella's" proposition that would need revising to fit the different localities, etc., but, as a whole, it is good, and I am pleased to know the subject has come up for comment. Susquehanna County has probably as much tuberculosis as any county in the State, and in looking over my books I find that about 25 per cent. of all cows tested are tuberculous, and not more than 25 per cent. of this number have been far enough advanced to show physical symptoms; the rest could have been kept as "Columella" suggests, with a great saving both to the owner and to the State.

About three years ago I tested a herd of nine thoroughbred Holsteins, four of which were imported from Holland at an expense of six hundred dollars each. Of the nine tested six were condemned, and we could allow the owner only fifty dollars per head. The idea of keeping them in quarantine was suggested to the owner, but he said he did not have a suitable place to keep them, and so they were destroyed. Five of this number could have been kept and a nice income been realized from them. This is a sample of what I have often found, hence I strongly favor this part of the suggestion. I have supervised the killing of a great many cattle that could have been (in part, at least) used for food and been more wholesome than some that is daily sold at our local markets; but if such a thing should be suggested in this section, and under our present mode of doing, the majority of farmers would have no faith in the test, and would pronounce the whole thing a fake. But if a slaughter-house were established for the purpose, and under the supervision of an inspector, the feeling would be entirely different.

Yours respectfully,

E. E. TOWER, D.V.S.

MONTROSE, PA., March 21, 1902.

